# Essays on Puerperal Fever and the Diseases Peculiar to Women, Selected from the Writings of British Authors Previous to the Close of the Eighteenth Century

**Published:** 1850-11

**Authors:** 


### ARTICLE V.

*Essays on the Puerperal Fever and other diseases peculiar to women, selected from the writings of British Authors previous to the close of the Eighteenth Century.*--Edited by FLEETWOOD CHURCHILL, M.D., M.R.I.A. Hon. Fellow of the College of Physicians of Ireland; Corresponding Member of the American National Institute; Hon. Member of the Philadelphia Medical Society, &c., &c, &c. Philadelphia: Lea & Blanchard, 1850; pp. 464; 8vo. (From the Publishers, and for sale by S. C. Griggs & Co., Chicago.)

This volume was prepared by the author at the request of the *Sydenham Society*, an association formed for the praiseworthy purpose of re-printing and preserving in an accessible form the writings of the old British Authors. Dr. Churchill has collected the essays of Denman, Hulme, Leake, Gordon, Clarke, and others, principally on Puerperal Fever; prefacing them by a very interesting and valuable history of the various epidemics of this disease, from the year 1746. Of course it is interesting to peruse the views of the old authors on a disease attended by such a frightful fatality as Puerperal Fever; and Dr. Churchill has conferred a favor on the profession, by assuming the editorial supervision of these essays. It is entertaining, as well as instructive, to note the gradual progress towards a correct knowledge of the pathology and treatment of this frightful scourge of women; and serves to remind us of our obligations to the fathers of medicine. As we shall probably hereafter have something more to say concerning this volume, we close our notice for the present by assuring our readers that they will find this volume a valuable one, both in a historical and practical point of view. M.

